# Cross cultural adaptation of the English version of the IOF-QLQ to Polish, to assess the health-related quality-of-life of patients after a distal radius fracture

**DOI:** 10.1186/s12955-015-0354-x

**Published:** 2015-09-29

**Authors:** Krzysztof A. Tomaszewski, Brandon Michael Henry, Jan Paradowski, Michał Kłosiński, Ewa Walocha, Joanna Golec, Ewa Kucharska, Zbigniew Dudkiewicz

**Affiliations:** Department of Anatomy, Jagiellonian University Medical College, 12 Kopernika St, 31-034 Krakow, Poland; Department of Orthopaedics and Trauma Surgery, 5th Military Hospital, Krakow, Poland; Department of Traumatology and Neuroorthopaedics, Rydygier Specialistic Hospital, Krakow, Poland; Department of Clinical Nursing, Institute of Nursing, Jagiellonian University Medical College, Krakow, Poland; Center for Medical Postgraduate Education, Jagiellonian University Medical College, Krakow, Poland; Department of Hand Surgery, Lodz Medical University, Lodz, Poland

**Keywords:** Distal radius fracture, IOF QLQ, Pilot-testing, SF-36, Validation, Wrist

## Abstract

**Background:**

A distal radius fracture (DRF) is a common injury that can cause significant pain and lead to a prolonged decrease in physical, emotional, and social functioning. In modern randomized clinical trials, assessing outcomes after a DRF, health-related quality-of-life (HRQoL) is a “must-be” endpoint. Additionally, HRQoL assessments are essential in the clinical decision-making process. The aim of this study to cross-culturally adapt the International Osteoporosis Foundation Quality of Life Questionnaire (IOF QLQ) for patients with a DRF to Polish.

**Methods:**

A standard forward-backward translation procedure and pilot-testing were used to prepare the Polish version of the IOF QLQ for use in this case–control study. Patients were eligible if they were between 18–80 years and were within 1–3 days after a non-comminuted DRF. The study group was gender and aged matched with healthy controls. All DRF patients filled out the Polish version of the IOF QLQ, the SF-36 and a demographic questionnaire. Assessment points were set as soon as possible after the fracture, 7 days, 6 weeks, 3, 6, 12, and 18 months after the fracture. Standard validity and reliability analyses were performed.

**Results:**

Ninety-seven patients (73 women – 75.3 %) with a mean age of 62.4 ± 7.1 years agreed to take part in the study. The control group consisted of 81 patients (60 women – 74.1 %) with a mean age 63.9 ± 8.2 years. No significant differences were found between the mean age of patients and controls (*p* = 0.19). Cronbach’s alpha coefficients showed positive internal consistency (0.79–0.89). The interclass correlations for the IOF QLQ domains and the overall score ranged from 0.85 to 0.92. Satisfactory convergent and discriminant validity of the IOF QLQ was seen.

**Conclusions:**

The Polish version of the IOF QLQ for patients with a DRF is a reliable and valid tool for measuring HRQoL. It can be fully recommended for use in clinical settings in the Polish population. When combined with the SF-36 the IOF QLQ allows to obtain a comprehensive HRQoL assessment in patients with a DRF.

## Background

Distal radius fracture (DRF) is one of the most common fractures, with an estimated annual incidence of 27 per 10,000 [1]. The incidence of DRF has a bimodal age distribution, peaking in pediatric and elderly populations [2]. In patients over the age of 65, DRF accounts for up to 18 % of all of fractures, with an increasing incidence due to the growing size of the elderly population [3, 4]. This peak in DRF incidence in the elderly is likely attributable to the increased risk of osteoporosis in postmenopausal women [5]. In older individuals, DRF often occurs due to low-energy force trauma, often due to a fall on a dorsally outstretched hand [4]. However in pediatric patients, where DRF accounts for up to 25 % of all fractures, DRF is often due to high-energy force trauma occurring through sport-related injuries, playing, or accidents [2, 4].

A DRF often causes short-term functional limitations, even after cast removal, such as a limited range of wrist and forearm motion, and decreased grip strength [2]. However, a DRF does not only cause pain and a loss of physical function, but also influences a patient’s emotional and social function, thus leading to a decrease in overall health-related quality-of-life (HRQoL) [6]. Furthermore, up to 21.2 % patients with a DRF may never fully recover from the injury, thus experiencing a permanent decrease in their HRQoL [2].

Additionally, a DRF may have severe, long-term consequences, such as the development of complex regional pain syndrome (CRPS). The reported incidence of CRPS in patients after a DRF ranges from 3.8 % to as high as 32.2 % [7, 8]. Furthermore, a decrease in a patient’s physical quality of life has been associated with an increase risk of development of CRPS [7].

A DRF often occurs early in the course of osteoporosis, while many patients are still employed and active [9]. As such, the social effects of a DRF extend beyond just the medical costs, resulting in loss of work hours, decreased occupational performance, loss of independence, and potential long-term disability [4, 10]. Thus, when considering the multiple treatment options available for a patient presenting with a DRF, it is important for clinicians to consider HRQoL advantages and disadvantages of each therapy, and tailor the treatment to each particular individual [11]. Additionally, HRQoL questionnaires could be used to assess patients in the first few months after fracture, when objective physical testing is not possible [9].

In modern randomized clinical trials assessing outcomes after a DRF, HRQoL is a “must-be” endpoint [12, 13]. Among the questionnaires used to assess recovery after a DRF are instruments specific for wrist fracture (eg. patient-rated wrist evaluation - PRWE), tools specific for the hand or wrist (eg. Michigan Hand Outcomes Questionnaire - MHQ), tools covering the function of the whole upper extremity (eg. disability of the arm, shoulder and hand questionnaire – DASH and its abbreviated version – quickDASH), as well as generic questionnaires such as the short form 36 (SF-36) or the EQ-5D [9, 14, 15]. While tests like PRWE and DASH may be useful for assessment of HRQoL for the purposes of clinical research, their use in clinical practice is considered by experts to be limited, lacking the valid information needed for improving the clinical decision-making process [15].

In 2010, the Working Group for Quality of Life of the IOF developed a questionnaire specific for HRQoL assessment in patients with wrist fracture - the IOF quality of life questionnaire for patients with wrist fracture (IOF QLQ) [9]. This new tool was found to be a reliable and responsive HRQoL questionnaire [9]. The latter, combined with the fact that Polish clinical practice lacks validated tools for HRQoL assessment in patients after a DRF, encouraged us to undertake this study.

The aim of this study was cross-culturally adapt and validate the IOF quality of life questionnaire for patients with a DRF in a Polish population.

## Materials and methods

### Study design

The study was designed as prospective multi-center study to assess the HRQoL in patients with a recent DRF as compared to age- and gender- matched control subjects. The patients were recruited between January 2013 and November 2014, from three hospitals in Poland - the 5th Military Hospital, Krakow, the Rydygier Specialistic Hospital, Krakow, and the University Clinical Hospital, Lodz. All patients with a DRF were followed for a period 18 months.

### Subjects

Patients were recruited into two groups. The study group comprised of patients with a radiographically confirmed DRF (treated surgically or non-surgically) and were selected by a qualified orthopedic surgeon (25+ years of experience). Patients were eligible if they were above 18 and below 80 years old and were within 1–3 days after the fracture. Exclusion criteria included lack of consent to participate in the study, inability to understand or complete the questionnaires, reoperation or remanipulation of the fracture, comminuted or pathological fractures, patients after polytrauma or patients with diseases with significant morbidity and having a severe impact on HRQoL, including cancer, congestive heart failure, and significant lung, renal, or liver diseases. The control group consisted of patients selected by an internal medicine physician (15+ years experience) who were reporting to the outpatient office for a routine annual checkup or for follow-up of mild disorders that did not impact the individual’s HRQoL such as mild hypertension or the common cold. Patients in the control group were chosen to gender and age match the subjects in the study group. Exclusion criteria included lack of consent to participate in the study, inability to understand or complete the questionnaires, severe arthritic changes, recent (up to 2 years) fractures and diseases having significant morbidity and a severe impact on HRQoL including cancer, congestive heart failure, and significant lung, renal, or liver diseases. The primary purpose of the control group in the study was to act as an estimate pre-fracture HRQoL level to compare the baseline HRQoL levels of patients with a DRF, and for assessment of the clinical validity of the IOF QLQ.

### Sample size calculation

Study sample size was based on the proposal of Tabachnick and Fidell [16], which states that in order to obtain reliable estimates, the number of observations should be 5–10 times the number of variables in the model. Thus, the required number of patients to conduct this study was between 60 and 120.

### Questionnaires

The SF-36 Health Survey is composed of 36 questions and standardized response choices, organized into eight multi-item scales: physical functioning (PF), role limitations due to physical health problems (RP), bodily pain (BP), general health perceptions (GH), vitality (VT), social functioning (SF), role limitations due to emotional problems (RE), and general mental health (MH). All raw scale scores are linearly converted to a 0 to 100 scale, with higher scores indicating higher levels of functioning or well-being. In this study we have used the freely available, pretranslated Polish version of the SF-36 [17].

The IOF QLQ for patients with wrist fracture (the translated Polish version can be seen in the Appendix) is composed of 12 questions scored on a 1 to 5 Likert scale. The questions form four domains – pain (question no. 1), upper limb symptoms (questions no. 2–4), physical function (questions no. 5–11), and general health (question no. 12). The scores on individual questions were summed up to form an overall score ranging from 12 to 60. This was later recalculated by linear transformation of raw scores into a score from 0 to 100, with 0 representing the best possible HRQoL [9]. Permission to use the IOF QLQ was obtained from the authors of the original study, and it should be noted that the original study is published open-access [9].

The Gartland and Werley Score was first designed by Gartland and Werley in 1951 [18]. The version used in this study follows the modification of Chun and Palmer [19]. This tool assesses wrist and hand function and is calculated by the medical doctor after completing a detailed patient history and physical examination. It assess wrist pain, function, motion, grip strength, fracture union, post-operative ulnar variance and whether any post-operative complications have occurred using both a subjective (patient reported) and objective (physical examination) evaluation. The minimum score is 27.5, and the maximum possible to obtain is 100 (representing best possible wrist function). Depending on the number of points scored the outcome is classified as excellent (no pain, disability, or limitation of motion), good (occasional pain, mild limitation of motion and function), or poor (severe pain, severe limitation of motion and function).

### Interview and examination procedure

The patients were recruited and informed about the details of the study during their visits at the emergency department or orthopedic outpatient clinics of the participating centers or during their stay at the orthopedic clinic. The interview and examination took place only after written, informed consent was obtained. The whole procedure was performed by qualified orthopedic surgeons.

For the study group, baseline patient characteristics were gathered using a standard questionnaire which enquired as to the gender and age of the patient, as well as the date, side (left/right, dominant/non-dominant), type of fracture and type of treatment (surgical or non-surgical – closed reduction and casting). Next the examining orthopedic surgeon, using the modified Gartland and Werley score [19], assessed the physical function of the wrist and hand of both upper limbs. After this, the patient was asked to complete the following questionnaires – the IOF QLQ and the SF-36.

Each patient was first examined as soon as possible after the fracture by a qualified orthopedic physician (usually on the same day the fracture occurred or within the next 24 h). Next, the patients were reexamined during each control visit at 7 days, 6 weeks, 3, 6, 12, and 18 months post fracture. Patients recruited to the control group were interviewed only once (at baseline). Full medical history was registered for each patient from their patient file.

During the interview process, assistance was offered to patients, if necessary, in two ways: 1) if the patient’s dominant extremity was fractured and the patient could not write, the interviewer marked the answers on the questionnaire for the patient and/or 2) if the patient did not have their reading glasses available, the interviewer would read the questions out loud to the patient. Interpreting questions or providing suggestions from the side of the interviewer was strictly forbidden.

A randomly chosen subset (based on a computer generated algorithm) of DRF patients (*n* = 30) completed one additional interview to assess the stability of the IOF QLQ. Patients in this subset completed one additional IOF QLQ assessment 7 days after completing the IOF QLQ at 6 months. All patients agreed to fill in the questionnaire for a second time.

Additionally, at the 12 month time point, patients in the DRF study group were asked two additional questions as a short qualitative survey: 1. Do you feel that in terms of your physical functioning you have returned to the pre-fracture level? and 2. Do you feel that in terms of your mental functioning you have returned to the pre-fracture level?. The available answer choices were yes, no, or I do not know.

### Questionnaire translation

**Fig. 1:**
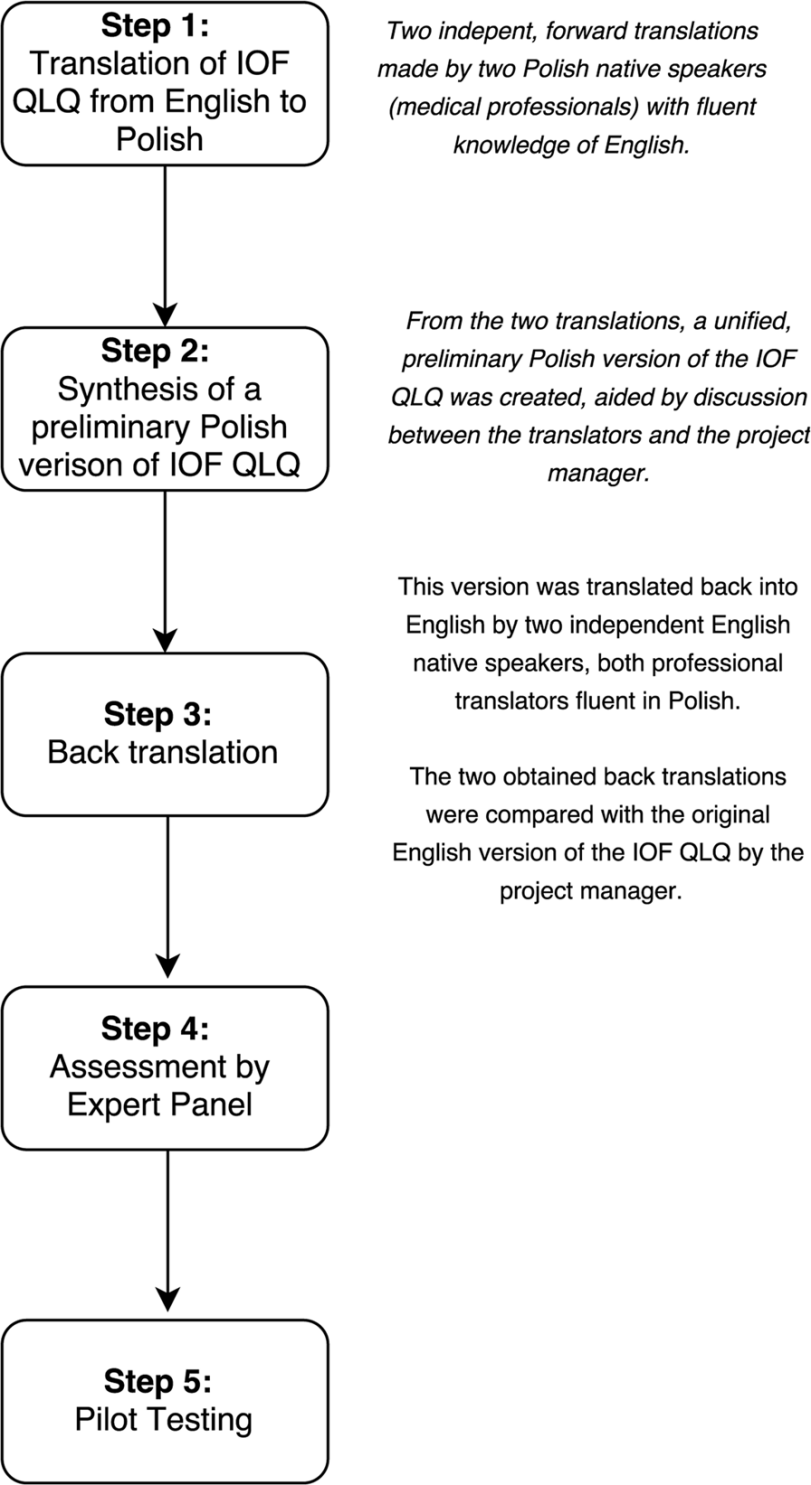
Flow chart of the process of cross-cultural adaptation of the IOF quality of life questionnaire for patients with wrist fracture

The translation was performed as per the European Organization for Research and Treatment of Cancer (EORTC) translation procedure [20] and per the guidelines developed by Beaton et al. [21] for cross-cultural adaptation of self-reported measures. The process of the questionnaire translation is outlined in Fig. 1.

In the next step, an expert panel was formed to confirm the semantic, idiomatic, conceptual, and experimental equivalence of the English and Polish questionnaire versions. The panel consisted of the project manager (orthopaedic surgeon), four translators who performed the forward and backward translations, 5 health care professionals (2 orthopaedics surgeons, a rheumatologist, a nurse, and a physical therapist), and an MSc in Polish studies. The final Polish IOF QLQ version for pretesting was prepared based on a consensus reached by the panelists.

It should be noted that the authors of this study have previous experience with studies concerning questionnaire validation in Polish populations [22, 23].

### Pilot-testing

The preliminary Polish version of the IOF QLQ was pilot-tested in a mixed group of 12 Polish patients with a DRF (mixed time since fracture occurred, different treatment methods). After reading and filling in the questionnaire, the patients were asked whether they found any of the questions difficult to answer, confusing, difficult to understand, upsetting or offensive. If a patient reported any of the above-mentioned problems, he or she was asked to suggest an alternative way to phrase the question. After completing the pilot testing phase, patients’ comments were analyzed by the project manager.

### Statistical analysis

Several pre-planned standard psychometric tests for quality of life studies, as outlined in the EORTC Module Development Guidelines, were conducted in our analysis [24]. Descriptive statistics (mean, standard deviation, percentage distribution) were used when appropriate to analyze demographic data. The Shapiro-Wilk test or the Kolmogorov-Smirnov test (as appropriate) were used to assess the distribution of the results. Statistical analysis was conducted using computer software Statistica 10.0 PL by StatSoft Poland (licensed to the Jagiellonian University Medical College). The significance level for all statistical tests was set at *p* < 0.05.

Convergent and discriminant validity were used to assess the construct validity of the domain structure of IOF QLQ. Both convergent and discriminant validity were tested using Spearman rank correlations, which were calculated between similar domains of the two questionnaires. Convergent validity was assessed by correlating each item with its own domain of the IOF QLQ [25–27]. Evidence of item convergent validity was defined as a correlation of 0.40 or greater between an item and its own domain (corrected for overlap). Discriminant validity was assessed by correlating each item with any other domain of the IOF QLQ [25, 26]. A scaling success for an item was seen when the correlation between an item and its own domain (corrected for overlap) was significantly higher (ie. two standard errors or greater) than its correlation with other scales [26, 27]. Calculating convergent and discriminant validity was only performed for “upper limb symptoms” and “physical function” domains because of the original structure of the questionnaire. As the other domains have only one item each, it is not possible to perform a correlation test.

Clinical validity was assessed using odds ratios. This assesses how well a specific domain is able to discriminate between groups of patients differing in clinical status [9]. In our analysis, clinical validity was assessed as study group vs. control group.

Reliability of the Polish version of the IOF QLQ was assessed by measuring the internal consistency and test-retest reliability. Cronbach’s alpha coefficient was calculated to assess the internal consistency. Internal consistency estimates of a magnitude of >0.70 were considered acceptable for group comparisons [27]. Cronbach’s alpha was calculated for “upper limb symptoms” and “physical function” domains as well as for the overall questionnaire score. Test-retest reliability (stability) of the IOF QLQ was assessed using interclass correlations (ICC) between baseline at 6 months and retest one week later. A correlation of >0.80 was considered ‘good’ [26, 27].

Assessment of the responsiveness of the scales to treatment was performed by comparing IOF QLQ scores at different time points of the study (baseline vs. 7 days, 6 weeks, 3, 6, 12, 18 months assessment) using the Mann–Whitney U test (due to the non-normal distribution of data). Similar analysis was used to compare mean IOF QLQ scores between subgroups of patients (male vs. female, dominant hand vs. nondominant hand being fractured, and surgical vs. nonsurgical treatment) in the study.

The acceptability of the IOF QLQ was assessed by the response rate, percentage of missing data, assistance and time needed to complete the questionnaire and details of items considered upsetting, confusing or difficult in the questionnaire [22, 23, 25]. This assessment was carried out in the same way as in the pilot-testing phase.

### Ethics, consent and permissions

The research protocol was approved by the Jagiellonian University Bioethics Committee (Registry No. KBET/176/B/2011 and KBET/187/B/2014). The study has been performed in accordance with the ethical standards laid down in the 1964 Declaration of Helsinki and its later amendments. Written, informed consent was obtained from each and every patient, of both the control group and the study group, before beginning the interview.

## Results

Since no significant differences were noted between the original version of IOF QLQ and the two back translations, the preliminary Polish version of the IOF QLQ was deemed ready for pilot-testing. During pilot testing, patients found all of the IOF QLQ questions acceptable and understandable. No language changes were needed to be made to the original translation.

**Fig. 2:**
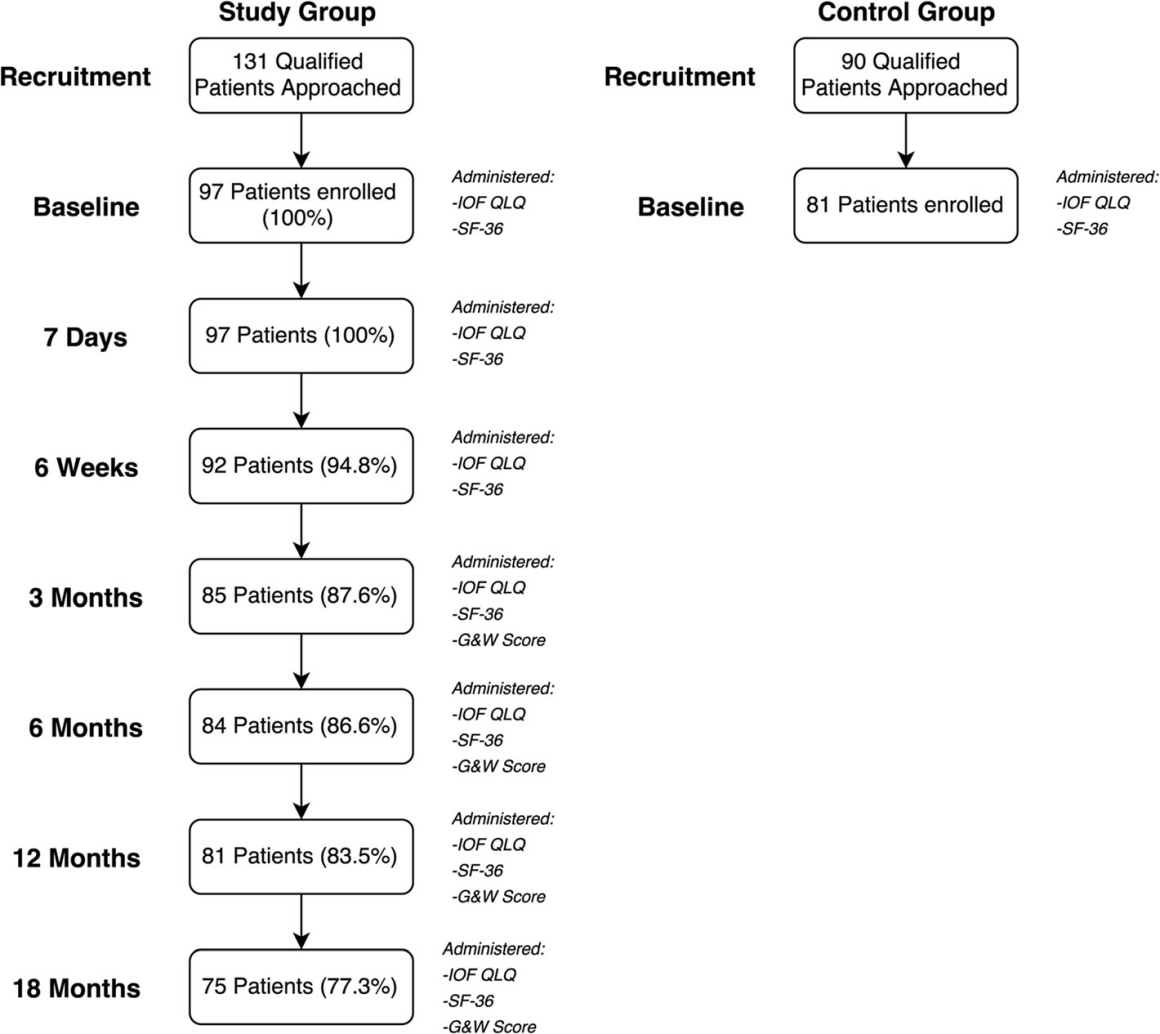
Flow chart of patient recruitment, assessment, and attrition to follow up throughout the study. IOF-QLQ - International Osteoporosis Foundation Quality of Life Questionnaire; G & W Score - Gartland and Werley Score

The flow of patients through the study is presented in Fig. 2. During the recruitment period, 131 patients with a DRF that qualified for the study were approached, of which 74 % (*n* = 97 patients, 73 women - 75.3 %) agreed to take part in the study. The mean age of the study group was 62.4 ± 7.1 years. A total of 34 qualified DRF patients (20 women - 58.8 %), with a mean age of 67.5 ± 7.5 years, refused to take part in the study.

During the recruitment of controls, a total of 90 qualified patients were approached to take part in the study, of which 90 % (*n* = 81 patients, 60 women – 74.1 %) agreed to participate. The mean age of the control group was 63.9 ± 8.2 years. A total of 9 qualified patients (3 women- 33.3 %), with a mean age of 59.3 ± 6.1 years, refused to participate in the study as controls.

There were no significant differences between the mean age of study group and the control group (*p* = 0.19). Table 1 presents baseline characteristics of the study group and attrition to follow-up. Additional demographic characteristics of the study group and the control group are presented in Table 2.

**Table 1:** Baseline characteristics of study participants

Feature		Study group (*n* = 97)
Age (mean ± SD) [years]		62.4 ± 7.1
Gender	Male (%)	24 (24.7 %)
	Female (%)	73 (75.3 %)
Side of fracture	Right (%)	39 (40.2 %)
	Left (%)	58 (59.8 %)
Fracture of extremity	Dominant (%)	40 (41.2 %)
	Non-dominant (%)	57 (58.8 %)
Type of fracture	Colles (%)	95 (97.9 %)
	Smith (%)	2 (2.1 %)
Type of treatment	Surgical (%)	33 (34 %)
	Non-surgical (%)	64 (66 %)

*SD* standard deviation, *n* number

**Table 2:** Further demographic characteristics of the study and control groups

Variable	Study group (*n* = 97)	Control group (*n* = 81)	p-value* (study vs. control group)
Education			
Elementary (%)	16 (16.5 %)	14 (17.3 %)	0.95
High School (%)	39 (40.2 %)	28 (34.6 %)	0.54
University (%)	42 (43.3 %)	39 (48.1 %)	0.63
Pre-fracture (study group) or current (control group) working status			
Employed (%)	54 (55.7 %)	46 (56.7 %)	0.99
Unemployed (%)	7 (7.2 %)	3 (3.7 %)	0.49
Retired/Pensioner (%)	30 (30.9 %)	27 (33.4 %)	0.85
Student (%)	6 (6.2 %)	5 (6.2 %)	0.76
Living			
Alone (%)	15 (15.5 %)	9 (11.2 %)	0.54
With partner or family (%)	70 (72.1 %)	62 (76.5 %)	0.62
With others (%)	12 (12.4 %)	10 (12.3 %)	0.84
Marital status			
Single (%)	9 (9.3 %)	13 (16.1 %)	0.25
Married (%)	81 (83.5 %)	64 (79.0 %)	0.35
Divorced (%)	7 (7.2 %)	4 (4.9 %)	0.75

*calculated using the Mann–Whitney U test

Overall, only 2.7 % of item responses were missing. Forty-eight interviewees (49.5 %) required assistance completing the questionnaires. Help was required mostly in order to mark answers on the questionnaire, due to the dominant extremity being fractured. The total time for completion of the questionnaires (excluding physical examination) was 17.2 ± 4.3 min without assistance, and 26.1 ± 5.5 min with assistance.

For the IOF QLQ “upper limb symptoms” domain, convergent validity was 0.68–0.77, discriminant validity was 0.22–0.38, and Cronbach’s alpha was 0.86. For the “physical function” domain convergent validity was 0.52–0.64, discriminant validity was 0.09–0.27 and Cronbach’s alpha was 0.79. Cronbach’s alpha calculated for the overall questionnaire score was 0.89. Cronbach’s alpha values, in case a specific item was deleted, are presented in Table 3.

**Table 3:** Cronbach’s alpha values, in case a specific item was deleted

Question number	“Upper limb symptoms” domain	“Physical function” domain	Whole questionnaire
Cronbach’s alpha value if all intended items present in domain/questionnaire			
-	0.86	0.79	0.89
Cronbach’s alpha value in case a specific item was deleted from the domain/questionnaire			
1	-	-	0.80
2	0.80	-	0.83
3	0.77	-	0.79
4	0.81	-	0.84
5	-	0.76	0.87
6	-	0.67	0.79
7	-	0.72	0.84
8	-	0.71	0.83
9	-	0.75	0.87
10	-	0.72	0.84
11	-	0.74	0.85
12	-	-	0.82

In the assessment of test-retest stability, ICCs for the IOF domains and the overall score ranged from 0.85 to 0.92, thus showing good repeatability of the scales.

Table 4 presents the discriminatory capacity of IOF QLQ questions and domains. The odds ratio for patients being categorized into the correct clinical status according to the Gartland and Warley Score were high and significant.

**Table 4:** The discriminatory capacity of IOF QLQ domains

	3 months (*n* = 85)		6 months (*n* = 84)		12 months (*n* = 81)		18 months (*n* = 75)	
IOF QLQ domains	Unadjusted OR (95 % CI)	Adjusted* OR (95 % CI)	Unadjusted OR (95 % CI)	Adjusted* OR (95 % CI)	Unadjusted OR (95 % CI)	Adjusted* OR (95 % CI)	Unadjusted OR (95 % CI)	Adjusted* OR (95 % CI)
Pain	1.63 (1.34–1.96)	1.81 (1.38–1.93)	1.50 (1.31–1.82)	1.52 (1.31–1.83)	1.49 (1.29–1.82)	1.50 (1.30–1.78)	1.46 (1.30–1.87)	1.50 (1.22–1.70)
Upper limb symptoms	1.37 (1.11–1.52)	1.40 (1.15–1.57)	1.35 (1.14–1.60)	1.35 (1.15–1.60)	1.33 (1.18–1.57)	1.35 (1.16–1.60)	1.34 (1.21–1.60)	1.38 (1.19–1.57)
Physical function	1.39 (1.26–1.63)	1.39 (1.25–1.60)	1.34 (1.15–1.45)	1.39 (1.16–1.48)	1.33 (1.12–1.41)	1.40 (1.19–1.48)	1.35 (1.13–1.42)	1.49 (1.21–1.45)
General health	1.18 (1.02–1.29)	1.20 (1.04–1.33)	1.16 (1.05–1.28)	1.18 (1.04–1.30)	1.21 (1.09–1.36)	1.21 (1.09–1.36)	1.21 (1.07–1.39)	1.25 (1.15–1.33)
Overall score	1.45 (1.30–1.71)	1.48 (1.31–1.73)	1.41 (1.28–1.67)	1.42 (1.26–1.67)	1.39 (1.22–1.65)	1.40 (1.22–1.68)	1.44 (1.24–1.61)	1.49 (1.25–1.69)

Odds ratios for being in the “poor” group vs. the “excellent” group according to the Modified Gartland & Werely score

*OR* odds ratio

*adjusted for age and gender. All OR given are with a *p* < 0.001

The IOF QLQ, the SF-36 and the Gartland and Warley Score mean scores, and their change over time is shown in Table 5. Changes over time in IOF QLQ domain scores in subgroups according to patients’ gender, treatment type and the dominant/non-dominant extremity being fractured are presented in Table 6.

**Table 5:** The IOF QLQ, the SF-36 and the Gartland and Werley score changes over time

Scale/Domain	Baseline *n* = 97	7 days *n* = 97	6 weeks *n* = 92	3 months *n* = 85	6 months *n* = 84	12 months *n* = 81	18 months *n* = 75	Control group *n* = 81
IOF QLQ								
Pain	69.7 (9.2)	58.1 (11.5) *p* < 0.0001	22.0 (7.4) *p* < 0.0001	14.6 (10.1) *p* < 0.0001	10.4 (6.7) *p* < 0.0001	7.5 (8.9) *p* < 0.0001	6.2 (9.1) *p* < 0.0001	0 *p* < 0.0001
Upper limb symptoms	44.7 (14.0)	51.3 (13.6) *p* = 0.001	30.1 (10.7) *p* < 0.0001	21.4 (9.2) *p* < 0.0001	14.4 (11.5) *p* < 0.0001	3.7 (14.2) *p* < 0.0001	3.1 (11.5) *p* < 0.0001	0 *p* < 0.0001
Physical function	82.8 (9.0)	81.3 (11.6) *p* = 0.55	58.9 (15.2) *p* < 0.0001	33.1 (7.0) *p* < 0.0001	16.6 (13.4) *p* < 0.0001	9.7 (10.5) *p* < 0.0001	10.1 (11.1) *p* < 0.0001	12.1 (6.6) *p* < 0.0001
General health	78.1 (13.7)	83.1 (9.9) *p* = 0.004	60.8 (12.2) *p* < 0.0001	37.2 (18.6) *p* < 0.0001	20.7 (11.3) *p* < 0.0001	7.0 (4.9) *p* < 0.0001	7.3 (5.1) *p* < 0.0001	0 *p* < 0.0001
Overall score	67.4 (10.2)	65.9 (11.0) *p* = 0.33	48.3 (10.8) *p* < 0.0001	27.8 (9.7) *p* < 0.0001	16.1 (12.0) *p* < 0.0001	7.6 (10.4) *p* < 0.0001	6.8 (10.0) *p* < 0.0001	4.3 (2.7) *p* < 0.0001
SF-36								
PF	59.7 (23.6)	57.1 (24.0) *p* = 0.45	66.4 (27.2) *p* = 0.07	73.6 (23.5) *p* = 0.0001	77.3 (20.1) *p* < 0.0001	84.1 (19.0) *p* < 0.0001	85.9 (16.8) *p* < 0.0001	87.0 (12.9) *p* < 0.0001
RP	15.1 (29.0)	27.4 (28.6) *p* = 0.003	38.0 (25.3) *p* < 0.0001	52.8 (21.2) *p* < 0.0001	59.3 (19.2) *p* < 0.0001	74.2 (21.4) *p* < 0.0001	81.5 (20.7) *p* < 0.0001	84.3 (24.1) *p* < 0.0001
BP	39.5 (27.1)	42.7 (24.1) *p* = 0.39	79.3 (19.3) *p* < 0.0001	79.8 (18.5) *p* < 0.0001	84.7 (17.2) *p* < 0.0001	87.5 (11.4) *p* < 0.0001	87.2 (12.1) *p* < 0.0001	88.7 (15.4) *p* < 0.0001
GH	63.5 (14.2)	65.2 (15.7) *p* = 0.43	74.1 (21.3) *p* = 0.0001	73.2 (20.2) *p* = 0.0002	75.7 (18.2) *p* < 0.0001	79.0 (15.6) *p* < 0.0001	78.3 (14.9) *p* < 0.0001	78.2 (14.4) *p* < 0.0001
VT	55.3 (16.9)	61.3 (26.2) *p* = 0.06	68.6 (20.4) *p* < 0.0001	71.0 (15.4) *p* < 0.0001	72.6 (13.1) *p* < 0.0001	73.3 (14.8) *p* < 0.0001	72.6 (12.8) *p* < 0.0001	73.7 (13.6) *p* < 0.0001
SF	51.7 (13.3)	51.4 (18.5) *p* = 0.90	69.2 (24.8) *p* < 0.0001	80.3 (22.5) *p* < 0.0001	88.1 (17.0) *p* < 0.0001	88.7 (20.5) *p* < 0.0001	88.9 (18.2) *p* < 0.0001	89.2 (18.3) *p* < 0.0001
RE	40.8 (17.0)	47.3 (20.6) *p* = 0.02	74.8 (17.6) *p* < 0.0001	83.4 (16.4) *p* < 0.0001	86.2 (19.8) *p* < 0.0001	90.4 (17.3) *p* < 0.0001	91.3 (21.4) *p* < 0.0001	90.0 (22.8) *p* < 0.0001
MH	67.4 (24.1)	70.3 (22.2) *p* = 0.39	78.8 (19.7) *p* = 0.0005	84.1 (16.4) *p* < 0.0001	82.0 (17.9) *p* < 0.0001	81.1 (22.6) *p* < 0.0001	82.3 (19.3) *p* < 0.0001	82.5 (19.9) *p* < 0.0001
Gartland & Werley score								
Excellent (%)	-	-	-	42.4 %	44.1 %	51.9 %	68.0 %	-
Good (%)	-	-	-	37.7 %	35.7 %	34.6 %	20.0 %	-
Poor (%)	-	-	-	19.9 %	20.2 %	13.5 %	12.0 %	-

Data presented as mean values ± (SD). The p-values are comparing baseline and specific time point scores or baseline and the control group

*SD* standard deviation, *n* number, *PF* physical functioning, *RP* role limitations due to physical health problems, *BP* bodily pain, *GH* general health perceptions, *VT* vitality, *SF* social functioning, *RE* role limitations due to emotional problems, *MH* general mental health

**Table 6:** The IOF QLQ score changes over time between patient subgroups

Assessment time-point	Mean (SD)		p-value*
	Surgical	Non-surgical	
0	68 (21.7)	67.1 (17.6)	0.83
1 week	60.9 (16.9)	68.5 (15.1)	0.027
6 weeks	36.7 (13.1)	54.3 (10.4)	<0.0001
3 months	21.4 (9.4)	31.1 (10.8)	<0.0001
6 months	10.7 (11.5)	18.9 (7.3)	<0.0001
12 months	5.9 (5.8)	8.5 (7)	0.07
18 months	5.3 (6.1)	7.5 (8.2)	0.18
	Males	Females	
0	59.2 (14.2)	70.1 (18.2)	0.009
1 week	59.5 (15.6)	68 (22)	0.08
6 weeks	50.1 (15.1)	47.7 (19.4)	0.58
3 months	21.7 (11.2)	29.8 (11.5)	0.003
6 months	11.5 (6.5)	17.6 (8.7)	0.002
12 months	5.8 (4.8)	8.2 (5.9)	0.07
18 months	5.3 (4.1)	7.1 (5.8)	0.16
	Dominant	Non-dominant	
0	72.1 (24.9)	64.1 (18.4)	0.07
1 week	70.9 (21.6)	62.4 (19.2)	0.04
6 weeks	60.6 (15.1)	39.7 (14)	<0.0001
3 months	39.9 (9.8)	19.3 (11.4)	<0.0001
6 months	22.5 (11.3)	11.6 (8.7)	<0.0001
12 months	10 (7.4)	5.9 (5.2)	0.0018
18 months	8.2 (6.6)	4.7 (4.3)	0.0021

The p-values are comparing the mean scores within each of the subgroups

*SD* standard deviation

*calculated using the Mann–Whitney U test

The majority of correlations between corresponding domains of the IOF QLQ and the SF-36 questionnaires were highly significant (*p* < 0.001), and all were strongly negative (*r* = −0.52 to *r* = −0.76) due to the difference in scoring of the two questionnaires. The correlations are presented in Table 7. The strongest correlations were noted between the “pain” and “bodily pain” scales (*r* = −0.76; *p* < 0.001), both “physical function” scales (*r* = −0.68; *p* < 0.001), and the “upper limb symptoms” and the “role limitations due to physical health problems” (*r* = −0.62; *p* < 0.001) scales.

**Table 7:** Correlation between domains of the IOF QLQ and the SF-36 questionnaires

Scale/Domain	IOF QLQ	IOF QLQ	IOF QLQ	IOF QLQ	IOF QLQ
	Pain	Upper limb symptoms	Physical function	General health	Overall score
SF-36 PF	*R* = −0.50	*R* = −0.53	*R* = −0.68	*R* = −0.59	*R* = −0.54
	*P* < 0.001	*P* < 0.001	*P* < 0.001	*P* < 0.001	*P* < 0.001
SF-36 RP	*R* = −0.55	*R* = −0.62	*R* = −0.59	*R* = −0.57	*R* = −0.48
	*P* < 0.001	*P* < 0.001	*P* < 0.001	*P* < 0.001	*P* < 0.001
SF-36 BP	*R* = −0.76	*R* = −0.49	*R* = −0.61	*R* = −0.53	*R* = −0.52
	*P* < 0.001	*P* < 0.001	*P* < 0.001	*P* < 0.001	*P* < 0.001
SF-36 GH	*R* = −0.55	*R* = −0.42	*R* = −0.44	*R* = −0.61	*R* = −0.57
	*P* < 0.001	*P* < 0.001	*P* < 0.001	*P* < 0.001	*P* < 0.001
SF-36 VT	*R* = −0.53	*R* = −0.39	*R* = −0.58	*R* = −0.63	*R* = −0.59
	*P* < 0.001	*P* < 0.001	*P* < 0.001	*P* < 0.001	*P* < 0.001
SF-36 SF	*R* = −0.44	*R* = −0.37	*R* = −0.40	*R* = −0.42	*R* = −0.39
	*P* < 0.001	*P* < 0.001	*P* < 0.001	*P* < 0.001	*P* < 0.001
SF-36 RE	*R* = −0.41	*R* = −0.29	*R* = −0.37	*R* = −0.36	*R* = −0.33
	*P* < 0.001	*P* < 0.05	*P* < 0.001	*P* < 0.001	*P* < 0.001
SF-36 MH	*R* = −0.39	*R* = −0.23	*R* = −0.30	*R* = −0.34	*R* = −0.37
	*P* < 0.001	P > 0.05	*P* < 0.05	*P* < 0.05	*P* < 0.001

The p-values indicate significance of correlation between two domains

*PF* physical functioning, *RP* role limitations due to physical health problems, *BP* bodily pain, *GH* general health perceptions, *VT* vitality, *SF* social functioning, *RE* role limitations due to emotional problems, *MH* general mental health

A total of 81 DRF patients took part in the qualitative survey at the 12 month time point. In response to the question “Do you feel that in terms of your physical functioning you have returned to the pre-fracture level?”, 88.9 % said yes, 8.6 % said no, and 2.5 % said I don’t know. In response to the question “Do you feel that in terms of your mental functioning you have returned to the pre-fracture level?”, 92.6 % said yes, while 7.4 % said no.

## Discussion

Despite the fact that DRF is a common fracture, the current literature lacks high-quality studies to define intervention or evaluation of treatment outcomes in patients with DRF [15]. Due to the high impact of a DRF on a patient’s physical, social, and emotional functioning, HRQoL is an essential measure for both evaluation of treatment outcomes in RCTs and as a tool in the clinical-decision making process [12, 13, 15]. While generic HRQoL measures such as the SF-36 may help to assess the overall HRQoL of a patient, and thus highlight important treatment-related issues, they have not been shown to be as responsive as more wrist specific assessments such as DASH and PRWE [14].

In this study, we have reported on the translation, pilot-testing, and validation of the IOF QLQ for patients with wrist fracture to confirm that this tool is an acceptable, psychometrically and clinically robust measure to collect HRQoL data in Polish patients with a DRF. To the best of our knowledge, this was the first study to translate, pilot-test and validate the IOF QLQ in Polish patients.

For a questionnaire to be acceptable for use in different countries, it must be first translated and appropriately validated in the target patient group. The results of our study indicate that the Polish version of the IOF QLQ demonstrates excellent agreement with the original language version [9]. The pilot-testing phase showed that the translated tool displays appropriate patient acceptability. This was confirmed later in the study by the low percentage of missing item responses.

During the recruitment phase, we noticed that a high percentage of patients (26.0 %) we planned to enroll in the study group refused to participate. The mean age of patients who refused to participate in the study was slightly older (5.1 years) than those who agreed to participate, suggesting that increased age was a factor in patient refusal. Furthermore, we debated whether to attribute the high rejection rate to the questionnaire format or to the fact that these patients had just suffered a painful fracture. This was later resolved when recruiting the control group, where patients were much more willing to take part in the study, thus showing that the questionnaire itself is an acceptable measure. The mean age of those refusing to participate as a control in the study was slightly younger (4.6 years) than those who agreed to participate as controls. We suspect that this was due to younger patients being more active and less willing to spend time participating in a study.

Data analysis revealed that both tested IOF QLQ domains as well as its overall score demonstrated appropriate Cronbach’s alpha values. This confirms the results from the international field study [9], proving IOF QLQs’ excellent internal consistency. Test-retest values in our study were found to be superior to those of the original study [9]. This might originate from the fact that Lips et al. [9] decided to assess IOF QLQ for stability at 3 months post fracture, with a 2 week period separating the test-retest interviews. At this time-point in the healing of a fracture, we suspected that 2 weeks may be enough time to change a patients’ HRQoL. This is why we choose to test-retest the IOF QLQ after 1 week at the 6 months post fracture time-point.

The results of known-group comparison demonstrated that the IOF QLQ is able to discriminate between patients differing in clinical status (study vs. control group), regardless of the time-point at which the assessment was carried out. Our findings confirm the discriminatory results reported by Lips et al. [9].

All of the four IOF QLQ domains, as well as its overall score, showed adequate responsiveness to change at almost every time-point, apart from the second assessment 7 days after the fracture. This early post-trauma period is characterized by a varying clinical course among patients, some presenting with more severe fractures than others, which may lead to significant short-term changes in HRQoL perception among different patients. Apart from demonstrating that the IOF QLQ responsiveness to change over a 12 month period is adequate in patients after a DRF, this study also depicted the history of HRQoL change in patients recovering after a DRF. The most significant HRQoL changes could be seen in the first 3 months after the fracture, which stands in agreement with other similar studies [9, 14, 28]. Between 12 and 18 months after a DRF, a patients’ HRQoL returned almost to the level displayed by the gender and age matched control group. The SF-36 scores proved a similar thesis, that HRQoL after a DRF returns to pre-fracture levels at 12 months after the injury. However the SF-36, supplemented by the qualitative assessment of patients mental function at 12 month time point, added information that the return to health was not only limited to physical function, but also included improvement of the mental aspect of HRQoL.

As could have been expected, patients with a fracture of the dominant extremity had a lower HRQoL, most probably because they were less able to care for themselves. This difference persisted throughout the study, including up to the last time point assessment of 18 months. This finding was also reported by Lips et al. [9], and confirms the face validity of IOF QLQ.

Patients receiving surgical treatment had a faster return to their pre-injury HRQoL. The HRQoL difference between surgically and non-surgically treated patients was most notable in the first 3–6 months after the DRF. However it is important to note that the HRQoL outcome after 18 months was similar in both groups. There were no important differences in overall IOF QLQ scores between genders, with women displaying only slightly lower HRQoL at 1 year post DRF.

The strong correlations in our study between similar IOF QLQ domains and the SF-36 scales is an important finding. Generic measures, like the SF-36, have some degree of specificity, however, it should be recognized that the term generic is relative and does not indicate universal applicability [29]. Due to the similarities between the two questionnaires, it would be possible to use the IOF QLQ and the SF-36 in conjunction to comprehensively assess HRQoL in patients with a DRF. To shorten the time needed for questionnaire completion, we suggest excluding the SF-36 questions pertaining to physical function, as this part would be covered by the IOF QLQ.

This study has a number of strengths. First, there was a large study group, with a well matched gender and age control group. Second, the follow-up attrition was minimal. Third, the assessment points were strictly adhered to. If a patient did not show up for a follow-up assessment, one of the study authors would call and invite the patient for a separately scheduled control visit. However, this study is not without limitations. First, there was a high rejection rate among patients we attempted to the recruit to the patient study group. Second, the inclusion/exclusion criteria may have biased the HRQoL score. Patients with comminuted fracture were excluded from the study, and their HRQoL would most likely be lower than the scores obtained in this study. However, this would most probably not have an impact on the overall psychometric properties of the IOF QLQ, but would likely influence HRQoL score changes over the follow-up period. We also did not compare the IOF QLQ to other “wrist-specific” instruments such as the DASH or the PRWE. However this was done intentionally, as this study aimed only at translating and validating the IOF QLQ, and comparing it to a generic HRQoL measure – the SF-36. We plan to compare the IOF QLQ to other “wrist-specific” tools in future studies.

## Conclusions

The Polish version of the IOF QLQ for patients with wrist fractures is a reliable and valid tool for measuring HRQoL in patients with a DRF. It can be fully recommended for use in clinical settings in the Polish population. When combined with the SF-36, the IOF QLQ allows to obtain a comprehensive HRQoL assessment in patients with a DRF.

## Appendix

### The final Polish translation of the IOF quality of life questionnaire for patients with wrist fracture

**Kwestionariusz IOF do oceny jakości życia u pacjentów ze złamaniem okolicy nadgarstka**

Wszystkie pytania odnoszą się do ostatniego tygodnia, z wyjątkiem pytania numer 12.

Proszę udzielić odpowiedzi na wszystkie pytania, niezależnie od strony złamania i kończyny dominującej.

1. Czy nadal odczuwa Pan/Pani ból w miejscu złamania przedramienia lub ręki?
  Wogóle; Trochę; Umiarkowanie; Dosyć mocno; Bardzo mocno.
2. Czy nadal odczuwa Pan/Pani drętwienie lub uczucie mrowienia w złamanym przedramieniu lub ręce?
  Wogóle; Trochę; Umiarkowanie; Dosyć mocno; Bardzo mocno.
3. Czy odczuwa Pan/Pani sztywność w złamanym przedramieniu lub ręce?
  Wogóle; Trochę; Umiarkowanie; Dosyć mocno; Bardzo mocno.
4. Czy niepokoi się Pan/Pani deformacją złamanego przedramienia?
  Wogóle; Trochę; Umiarkowanie; Dosyć mocno; Bardzo mocno.
5. Czy jest Pan/Pani w stanie umyć lub wysuszyć suszarką swoje włosy?
  Bez trudności; Z niewielką trudnością; Z umiarkowaną trudnością; Z dużą trudnością; Nie jestem w stanie wykonać tych czynności.
6. Czy jest Pan/Pani w stanie przekręcić klucz w zamku lub odkręcić pokrywkę słoika?
  Bez trudności; Z niewielką trudnością; Z umiarkowaną trudnością; Z dużą trudnością; Nie jestem w stanie wykonać tych czynności.
7. Czy ma Pan/Pani problemy z wykonywaniem pracy zawodowej lub prac domowych?
  Bez trudności; Z niewielką trudnością; Z umiarkowaną trudnością; Wymagam pewnej pomocy; Nie jestem w stanie wykonać tych czynności.
8. Czy ma Pan/Pani problemy z pisaniem odręcznie lub pisaniem na maszynie/klawiaturze komputerowej?
  Bez trudności; Z niewielką trudnością; Z umiarkowaną trudnością; Z dużą trudnością; Nie jestem w stanie wykonać tych czynności.
9. Czy jest Pan/Pani w stanie korzystać z własnego środka transportu tj. prowadzić samochód lub jeździć na rowerze?
  Bez trudności; Z niewielką trudnością; Z umiarkowaną trudnością; Z dużą trudnością; Nie jestem w stanie wykonać tych czynności.
10. W jakim zakresie Pana/Pani złamane przedramię przeszkadzało w zajęciach w trakcie ostatniego tygodnia?
  Wogóle; Trochę; Umiarkowanie; Dosyć mocno; Bardzo mocno.
11. Czy potrzebuje Pan/Pani pomocy ze strony przyjaciół lub krewnych z powodu złamania przedramienia?
  Nie potrzebuję; 1 dzień w tygodniu lub mniej; 2–3 dni w tygodniu; 4–6 dni w tygodniu; Codziennie.
12. Czy w Pana/Pani ocenie, Pana/Pani jakość życia, w przeciągu ostatnich 3 miesięcy pogorszyła się ze względu na złamanie przedramienia?
  Wogóle; Trochę; Umiarkowanie; Dosyć mocno; Bardzo mocno.
